# Assessment of Meniere's disease from a radiological aspect – saccular otoconia as a cause of Meniere's disease?

**DOI:** 10.3109/00016489.2012.680980

**Published:** 2012-09-21

**Authors:** Hideo Yamane, Kishiko Sunami, Hiroyoshi Iguchi, Hiramori Sakamoto, Toshio Imoto, Helge Rask-Andersen

**Affiliations:** ^1^Department of Otorhinolaryngology, Osaka City University Graduate School of Medicine, Osaka, Japan; ^2^Department of Otorhinolaryngology, Uppsala University Hospital, Uppsala, Sweden

**Keywords:** Saccular duct, saccule, endolymphatic hydrops, endolymphatic duct, CT image, bilaterality

## Abstract

**Conclusion:**

Significant reduced visualization of the reuniting duct (ductus reuniens; RD), saccular duct (SD) and endolymphatic sinus (ES) in Meniere's disease (MD) compared with normal control ears on three-dimensional (3D) CT imaging suggests the blockage of endolymphatic flow there with radiodense substances, which may be explained by dislodged otoconia from the saccule. These structures could be involved in the pathogenesis of MD.

**Objective:**

This study was designed to visualize and assess the RD, SD and ES in patients with MD using 3D CT.

**Methods:**

Sixty-two patients with a definite diagnose of unilateral MD, based on criteria proposed by the Committee on Hearing and Equilibrium of the American Academy of Otolaryngology-Head and Neck Surgery (AAO-HNS), were compared with contralateral ears and normal controls (26 ears) using 3D CT. The RD, SD and ES were scrutinized for patency on 3D CT images.

**Results:**

MD ears showed loss of continuity of the RD, SD and ES based on evaluation of 3D CT images, and differed significantly from normal healthy control ears (*p* < 0.01).

## Introduction

The etiology of Meniere's disease (MD) remains a riddle in spite of many studies. Most such studies are based on the idea of endolympatic hydrops [1,2]. Even though the existence of a ‘longitudinal flow’ has been challenged experimentally by some researchers in recent years, a disturbance of endolymph circulation within the duct system is generally agreed to be an important factor for generation of endolymphatic hydrops in MD [3,4].

We previously reported on the visualization of the reuniting duct (RD) and saccular duct (SD) and endolymphatic sinus (ES) of the human inner ear by analysing their bony grooves using three-dimensional (3D) cone beam CT images [5,6]. As the RD, SD and ES each lodge onto these bony grooves, analysis of the grooves can yield information on the condition of the RD, SD and ES. Using this strategy, we reported that MD patients, defined as grade 3 by the criteria of MD from the Committee on Hearing and Equilibrium of the American Academy of Otolaryngology-Head and Neck Surgery (AAO-HNS), showed significant discontinuity of the bony groove of the RD in comparison with normal ears. This discontinuity suggests that the RD may be blocked by a radiodense substance [7], and this substance could represent dislodged saccular otoconia.

The saccule contains two ducts, the RD and the SD. They are believed to convey endolymph or pressure release between the endolymphatic compartments. Investigating these two ducts may be crucial when analysing MD patients from the viewpoint of patent ‘longitudinal flow’. In the present study we report on the incidence of patency of these two ducts and the ES in MD patients compared to controls.

## Material and methods

### Subjects

We studied 62 patients (29 males and 33 females; mean age 55.5 years, range 25–86 years) with diagnosed unilateral MD as defined by the criteria of the Committee on Hearing and Equilibrium of the AAO-HNS. The affected ears of patients with MD were compared with the non-affected contralateral ears and the bilateral healthy ears in 13 volunteers (6 males and 7 females, mean age 57.9 years, range 35–79 years). Four frequencies at 0.5, 1, 2 and 4 kHz were used to calculate pure tone averages. Although a 3 kHz threshold is included in the four pure tone averages used for definition of MD by AAO-HNS, 3 kHz is not commonly used in Japan, and 4 kHz was substituted for 3 kHz in this study.

Approval for this study was obtained from the ethics committee of Osaka City University Graduate School of Medicine.

### Analysis of CT image

The temporal bones were examined using 3D cone beam CT (3D Accuitomo; J. Morita Mfg Corp., Kyoto, Japan) using the same conditions as reported previously [5,6]: 80 kV; 6 mA; voxel, 0.125 mm × 0.125 mm × 0.125 mm; slice thickness, 0.5 mm. The CT images were taken in a region of interest of diameter 6 cm and height 6 cm. Reconstructed 3D images of the inner ear were obtained by rendering software (IVIEW) in perspective view with a viewing angle of 15° and 0.25 mm voxel size (0.25 mm × 0.25 mm × 0.25 mm).

We previously reported how to reduce artefacts due to rendering effects [5,6] by comparing the findings of a cadaver temporal bone and its 3D CT image using several landmarks. In the present investigation of the image patterns of the RD, SD and ES, we adopted an additional image in which the common crus formed a horizontal line, so rotation would not change the view from directly above.

**Figure 1.**
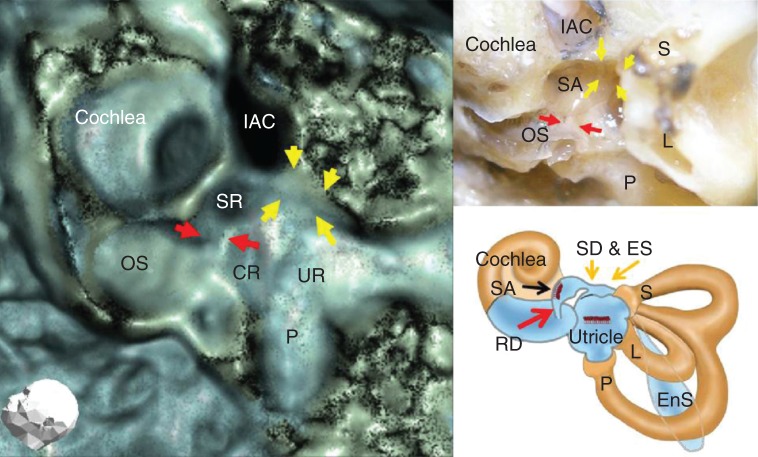
3D CT image of the reuniting duct (RD), saccular duct (SD) and endolymphatic sinus (ES) of a healthy volunteer's ear (left), cadaver's ear (upper right), and schematic view (lower right). All these views are left ears. Red arrows show the RD and yellow arrows show the SD and ES. CR, cochlear recess; EnS, endolymphatic sac; IAC, internal auditory canal; L, lateral semicircular canal; OS, osseous spiral lamina; P, posterior semicircular canal; S, superior semicircular canal; SA saccule; SR, saccular recess; UR, utricular recess.

**Figure 2.**
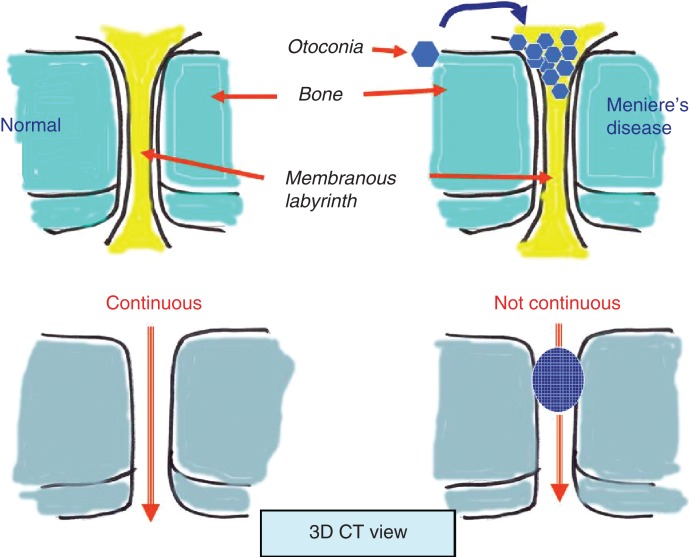
Drawings demonstrating the patency of the reuniting duct (RD), saccular duct (SD) and endolymphatic sinus (ES) on 3D CT images. When otoconia are dislodged into the RD, SD or ES, they lose continuity of the surface of their bony grooves and become vague on 3D CT images.

We evaluated the patency of the grooves of the RD, SD and ES (Figure 1). The patency of the RD was assessed on the orifice of the saccule to the RD based on our previous report [7]. The patencies of the SD and ES were analysed in the same manner by assessing the continuity of their bony grooves [6]. In this study the appearances of the grooves of the RD, SD and ES as not fully recognized was defined as closed and any other state was defined as open to avoid the weak point of the subjective visual evaluation of 3D CT images (Figure 2).

Additionally, we examined CT images of a cadaver with a small piece of muscle or small amount of calcium carbonate on the bony grooves of SD and ES to assess changes in the CT images, as in our previous report [7].

### Statistical analysis

The 62 patients were analyzed in comparison with the normal subjects. The incidence of abnormal images of the bony grooves of those portions in affected ears in MD patients was compared with the non-affected contralateral ears and control volunteer ears and analysed by Yates 2 × 2 chi-squared test.

## Results

### Findings of RD aspects

**Figure 3.**
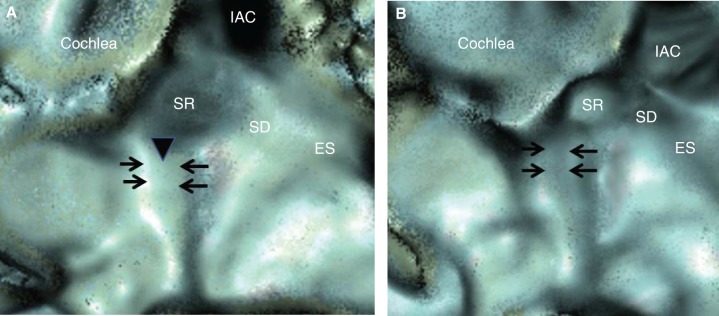
Representative views of the left ear of a patient with Meniere's disease (MD) (A) and a volunteer's healthy ear (B). (A) Although the outline of the bony groove of the reuniting duct (RD) (arrows), saccular duct (SD) and endolymphatic sinus (ES) can be traced, their luminal spaces seem to be occupied by a dense, bony substance. (B) On the other hand, those of the volunteer's healthy ear do not seem to be occupied by such a substance, and the luminal spaces maintain continuity. The arrowhead shows the discontinuity of the groove of the RD (arrows) in the MD patient. IAC, internal auditory canal; SR, saccular recess.

**Figure 4.**
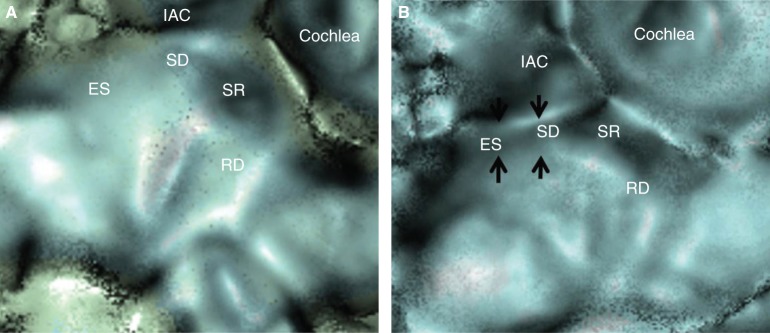
Representative views of the right ear of a patient with Meniere's disease (MD) (A) and a volunteer's healthy ear (B). (A) Both the bony grooves of the reuniting duct (RD) and the endolymphatic sinus (ES) are fully occupied by a dense, bony substance and it is hard to trace their luminal spaces, but that of the saccular duct (SD) is not fully occupied in the MD patient. (B) The bony grooves of the RD, SD and ES of the volunteer's healthy ear are not occupied and maintain their luminal spaces. The bony grooves of the SD and ES show continuous images (arrows). IAC, internal auditory canal; SR saccular recess.

**Figure 5.**
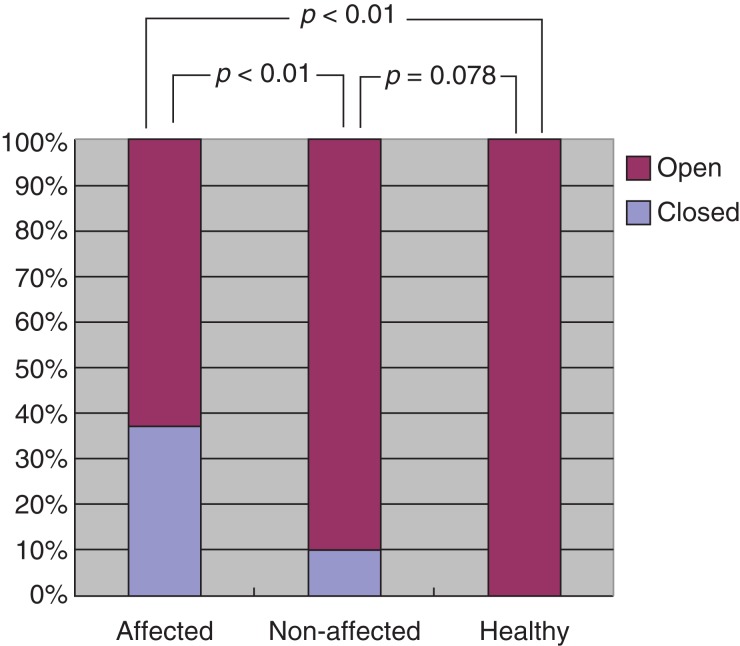
Distribution pattern of the reuniting duct (RD) in affected and non-affected ears of patients with Meniere's disease (MD) and healthy ears of volunteers.

Figures 3 and 4 show representative views of different patencies of the RD, SD and ES. Of the ears on the affected side of MD patients (Figures 3A and 4A), 37% (23/62)) had closed RD compared with 9.7% (6/62) of the ears on the non-affected side (Table I). None (0/26) were closed in the normal group (Figures 3B and 4B). There were significant differences between the ears on the affected side of MD patients and normal ears, and between the affected ears and the non-affected ears (*p* < 0.01) (Figure 5). There was no significant difference between non-affected ears of MD patients and normal ears (*p* = 0.078).

**Table I.** Status of the reuniting duct (RD), saccular duct (SD) and endolymphatic sinus (ES) in 62 patients with Meniere's disease (MD) and 13 healthy volunteers.

			Patency of groove		
Region	Ear		Closed	Open	Total
RD	MD	Affected	23	39	62
		Non-affected	6	56	62
	Healthy		0	26	26
SD	MD	Affected	32	30	62
		Non-affected	12	50	62
	Healthy		0	26	26
ES	MD	Affected	40	22	62
		Non-affected	11	51	62
	Healthy		0	26	26

Open, patent; closed, not patent.

### Findings of SD aspects

**Figure 6.**
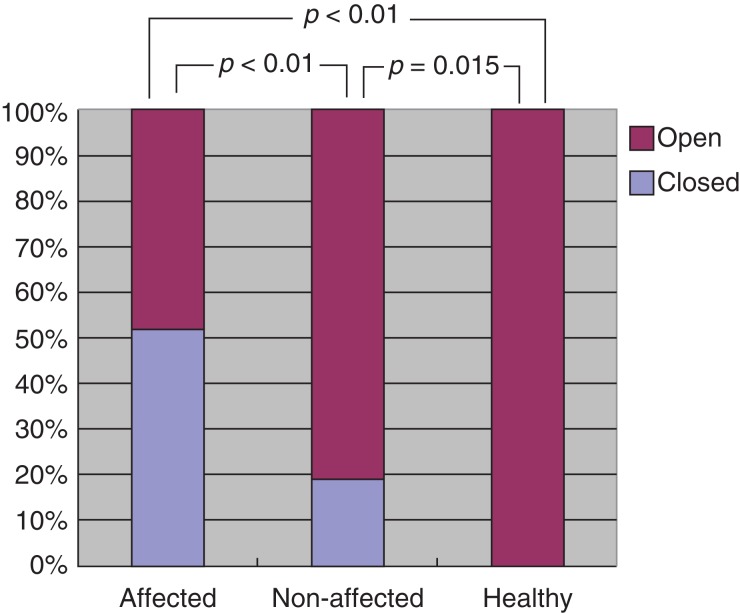
Distribution pattern of the saccular duct (SD) in affected and non-affected ears of patients with Meniere's disease (MD) and healthy ears of volunteers.

The patencies of the affected and non-affected ears of MD were 51.6% (32/62) and 16.1% (12/62), respectively (Table I). In contrast, all the SDs (26/26) were patent in the healthy ears (Figures 3B and 4B). There were significant differences between the affected ears and healthy ears and between the affected ears and the non-affected ears (*p* < 0.01). There was a slight but significant difference between the non-affected ears and healthy ears (*p* = 0.015) (Figure 6).

### Findings of ES aspects

**Figure 7.**
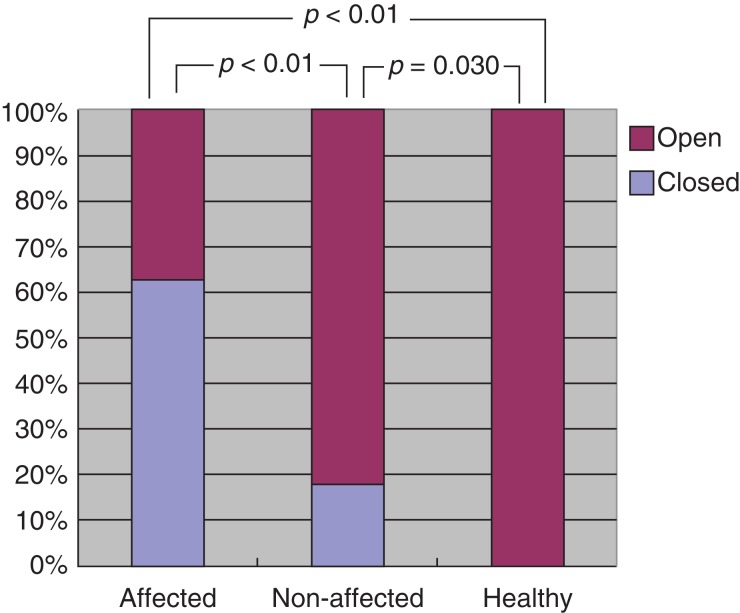
Distribution pattern of the endolymphatic sinus (ES) in affected and non-affected ears of patients with Meniere's disease (MD) and healthy ears of volunteers.

The distribution pattern of the ES resembled that of the SD. The patencies of the affected ears and non-affected ears of MD patients were 64.5% (40/62) and 17.7% (11/62), respectively (Table I). All the ESs (26/26) were patent in the healthy ears (Figures 3B and 4B). There were significant differences between the affected ears and healthy ears and between the affected ears and the non-affected ears (*p* < 0.01). There was a slight difference between the non-affected ears and healthy ears (*p* = 0.030) (Figure 7).

### Findings in the SD and ES of a cadaver treated with CaCO_3_ and soft tissue fragments

**Figure 8.**
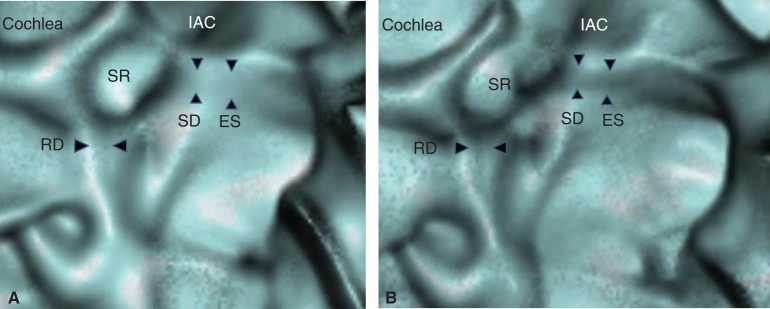
3D CT views of a cadaver temporal bone. The bony grooves underlining the saccular duct (SD) and the endolymphatic sinus (ES) treated with CaCO_3_ (A) appear vague compared with those treated with muscle fragments (B). Note the difference in continuity of the bony grooves (arrowheads). IAC, internal auditory canal; RD, reuniting duct; SR, saccular recess.

The bony grooves of the SD and ES treated with CaCO_3_ appeared vague (Figure 8A), but those with muscle fragments were not affected (Figure 8B).

## Discussion

We could demonstrate differences in visualization or patency of the RD, SD and ES between the affected ears of MD patients and normal ears, suggesting that the RD, SD or ES in MD ears may be partly or entirely occluded compared with healthy ears. The reduced visualization of the RD, SD and ES suggests that these ducts were involved as lesions of MD.

Debris, pathogens or unusual substances in the inner ear can be transported in these narrow ducts and become obstacles to the pathway of longitudinal flow of endolymph resulting in endolymphatic hydrops.

We speculate that a reasonable explanation for this reduced visualization may be an occlusion caused by dislodged otoconia from the saccule. This is based on experimental findings from cadavers showing that the bony groove appears vague when covered with CaCO_3_ but not when covered with muscle fragments. The data from the present and previous studies [7] suggest that the occlusion may be situated between the saccule and the RD or the SD and the ES.

Saccular otoconia could be dislodged by various causes such as aging as the natural fate, infection, disturbance of blood circulation, trauma, etc., and the dislodged otoconia from the saccule can disperse into the surrounding membranous labyrinth [8,9].

If otoconia fall into such a narrow pathway, the RD to the cochlea or the SD and ES to the endolymphatic duct, they could disturb the endolymphatic flow.

Although saccular otoconia are more susceptible than those of the utricle [10], the incidence of MD patients is not so high in comparison with benign paroxysmal positional vertigo (BPPV) possibly caused by dislodged utricular otoconia [11]. We do not have conclusive data proving this. However, some speculation from our present results may answer this question. The risk of saccular otoconia falling into the RD may not be so high because of its narrowness, even if it is near the saccular otolithic membrane.

The longitudinal endolymph may work to oppose saccular otoconia falling into the RD. On the other hand, the SD and ES are in the path of the anterograde endolymph system. Therefore, the SD (51.6%) and ES (64.5%) may be more occluded than the RD (37%). The character of MD may be due to the rate of occlusion of these narrow ducts.

Histological studies reported that the endolympatic duct (ED) was obstructed with fibrous tissue, basophilic substance or bone in MD [12] and a radiological study also showed narrow and poor development of the ED in MD [13]. The lesions of the ED may have a similar pathology to those of the SD and ES because all these regions are connected as successive membranous pathways, which could support our hypothesis that dislodged saccular otoconia could be a cause of MD. However, more precise investigation of these areas of the human temporal bone in MD is necessary.

Although the non-affected ears in patients with MD showed a similar RD pattern to the affected ears, the similarity was not statistically supported. On the other hand, there were slight differences in the patency of the SD and ES between the non-affected ears and healthy ears. This tendency of occlusion in the SD and ES of the non-affected ears of MD patients suggests that the contralateral non-affected ears may also be challenged but functionally compensated. Earlier acoustic biasing experiments have shown that incipient endolymphatic hydrops may exist in the healthy contralateral ear in MD.

Bilateral engagement of MD varies according to different authors. The longer the duration of the disease the more frequent is the incidence of bilateral disease [14]. In a recent German study where the long-term course of MD was revisited, as many as 35% of subjects suffered from MD in both ears after 10 years and 47% after 20 years [15]. Some studies show a low bilateral incidence (5%) while audiometric low-tone changes occur more frequently (16%) [16]. Also, the modulation depth was found to be significantly reduced in the contralateral non-symptomatic ears of MD patients using low-frequency masking to diagnose endolymphatic hydrops [17]. This has led to the conception that MD is basically a bilateral disease with differing expression in the two ears. In addition, histopathological changes have been described to a large extent in the contralateral temporal bone in patients with unilateral MD [18]. Genetic alterations such as single nucelotide polymorphisms have been described in patients with bilateral MD [19]. The onset of MD has also been found to be earlier when both ears are affected [20]. Although we could not verify signs of bilateralism in our patients with MD in the present unilateral cases, the unilateral group may have included some bilateral cases. This is supported by our 3D CT findings. However, bilaterality of MD must be fully investigated in a future study.

**Figure 9.**
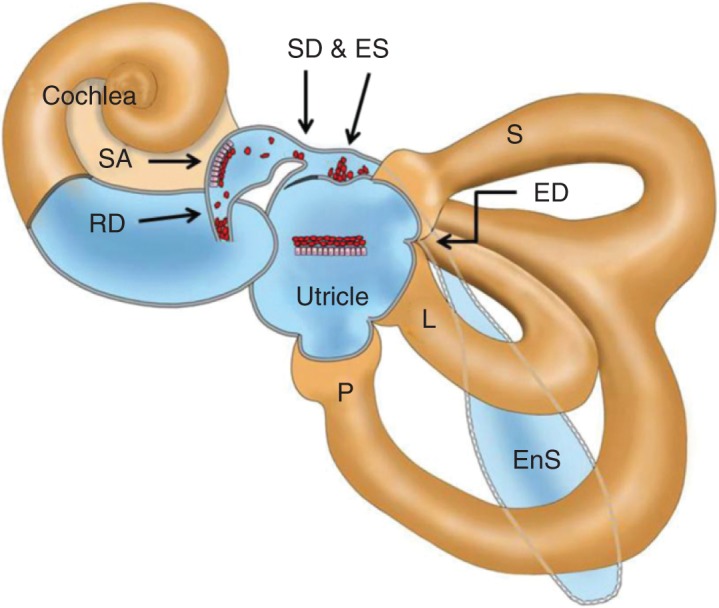
Hypothetical view of blockage of the reuniting duct (RD), saccular duct (SD) and endolymphatic sinus (ES) by dislodged otoconia from the saccule in patients with Meniere's disease (MD). ED, endolymphatic duct; EnS, endolymphatic sac; L, lateral semicircular canal; P, posterior semicircular canal; S, superior semicircular canal; SA, saccule.

We hypothesize that MD is a pathological condition brought about by saccular dislodged otoconia due to several causes, obstructing the narrow paths of the endolymph, based on the etiology of BPPV caused by dislodged otoconia from the utricle (Figure 9). Although we must investigate many factors of MD and the mechanism of MD attack in the future, the opening of these narrow paths could be one effective therapy for MD.

We evaluated the patency of the RD, SD and ES by 3D CT imaging, reflected by their bony grooves and not the actual ducts and ES. We thus obtained no definitive data on the degree of obstruction using the present 3D CT images.

In addition, the differences on 3D CT images among the various stages of MD that are categorized by degree of hearing deterioration must be investigated in the future.
